# Editorial: Identification of Multiple Targets in the Fight Against Alzheimer's Disease

**DOI:** 10.3389/fnagi.2020.00169

**Published:** 2020-06-16

**Authors:** Patrizia Giannoni, Silvia Fossati, Elena Marcello, Sylvie Claeysen

**Affiliations:** ^1^EA7352 CHROME, University of Nîmes, Nîmes, France; ^2^Alzheimer's Center at Temple (ACT), Lewis Katz School of Medicine, Temple University, Philadelphia, PA, United States; ^3^Department of Pharmacological and Biomolecular Sciences, Universitá Degli Studi Di Milano, Milan, Italy; ^4^IGF, Univ Montpellier, CNRS, INSERM, Montpellier, France

**Keywords:** inflammation, multi-interventions, neurovascular unit, microbiome, hormones, APP, beta-amyloid

## Introduction

This Research Topic is a collection of 20 articles that depict a broad representation of the most impactful advances in Alzheimer's disease (AD) comprehension and therapeutic openings. As it clearly emerges from recent literature, AD is a complex pathology with many different phenotypes and heterogeneous clinical settings. Although in a minority of cases genetic mutations have been linked to its development, for the vast majority of AD patients the triggering event remains to be elucidated. Main hallmarks such as amyloid beta (Aβ) plaques and neurofibrillary tangles have been identified, but understanding their exact role on cognitive consequences, timing of appearance, mechanisms of toxicity, and interplay required years of studies, with many questions still remaining unanswered. To note, the events possibly driving the development of such pathological signs are diverse and much more numerous than expected. For this reason, scientists have been directing their efforts to improving the understanding of the pathways involved in the toxicity mechanisms observed. Indeed, only a profound knowledge of the full process bringing to the different pathological phenotypes will help in figuring out effective treatments and/or preventive actions. With this concept in mind we have developed the present topic, which aims at giving a far-reaching picture of our actual knowledge, from the etiology of AD to mechanistic insights and possible new targets of intervention. Authors present their latest discoveries on a variety of breakthrough AD-related subjects such as inflammation, microbiome, hormones, Aβ production and catabolism, and neurovascular unit (NVU) alterations. Notably, the importance of reliable biomarkers as well as the paramount role of global approaches for the treatment/prevention of AD (see multi-interventions), are granted and finely discussed. We present here a summary of the main fields covered by this collection that we believe will constitute critical hints for future research development.

## APP Metabolism

According to the amyloid hypothesis, the increase in brain Aβ levels is a central event in AD pathogenesis. The soluble Aβ oligomers trigger synaptic dysfunction and, thereby, early cognitive deficits. Synaptic failure can occur also in the olfactory bulb leading to olfactory deficits that can be detected in many AD patients. Li et al. revealed a critical mechanism underlying olfactory dysfunction in AD showing that Aβ deposition induces morphological and functional changes in the synapses of the olfactory processing network in APP/PS1 mice during aging.

Aβ results from the proteolysis of the Amyloid Precursor Protein (APP). β-secretase BACE1 and γ-secretase concerted action on APP releases Aβ peptide, while the α-secretase ADAM10 (A Disintegrin and metalloproteinase domain-containing protein 10) cleaves APP within Aβ sequence, thereby preventing its generation. Promoting the α-cleavage of APP not only precludes the formation of Aβ but also increases the release of the neuroprotective sAPPα fragment. This can be achieved by acitretin, an activator of the α-secretase ADAM10, as assessed by dos Santos Guilherme et al. in the 5XFAD mouse model and in humans.

Even though APP metabolism has been studied in detail, it is still a central question in AD. Haytural et al. highlighted the technical concerns related to a potentially non-specific western blotting band at the expected molecular weight of APP-derived fragments, which calls for precaution when analyzing proteins of this size in human brain tissue. García-González et al. emphasized the importance of the comprehension of APP processing since APP can be a common substrate for several proteases. Among them, MT-MMPs (Membrane-type Metalloproteinases) are at the crossroads of pathological events involving not only amyloidogenesis, but also neuroinflammation and synaptic failure, thus opening up new research perspectives in AD.

## Neuroinflammation

Neuroinflammation is definitely a hot-topic and one of the most studied fields in AD and related dementias in recent years. As summarized in the review by Hemonnot et al., many aspects of inflammation are still not completely understood. Microglial cells, main players in inflammatory reactions, are complex and heterogeneous cells. Several activation states exist and a fine-tuned characterization of AD stages is needed in order to design effective preventive and/or curative microglial-targeting interventions. One main aspect that slows down our full understanding of neuroinflammation is represented by the numerous mechanisms that might contribute to its development and progression. Hemonnot et al. presented an analysis of recent GWAS studies and described major genes implicated in microglial cells regulation, such as Apolipoprotein E (APOE) and TREM2 (triggering receptor expressed on myeloid cells 2). Finally, the authors proposed the purinergic signaling as one possible target of intervention for microglial state modulation. Another potential pharmacological target was evidenced by Adorni et al., that highlighted the role of proprotein convertase subtilisin/kexin type 9 (PCSK9) not only in cholesterol homeostasis, but also in neuroinflammation. Finally, Cerovic et al. evidenced in their review the strong link between gut microbiota (GM) and neuroinflammation development and exacerbation. The limitations of experimental protocols constitute a significant obstacle to which researchers are trying to find innovative solutions. Indeed, laboratory models only partially reproduce the complex reality of human AD inflammation. This is true for the microbiome status of animals living in animal facilities, which is far from the “real life” status, transgenic models (Hemonnot et al.) that focus in most cases on specific and limited AD features, and many other constraints, such as the fact that marked immunological differences exist between animals and humans as well as between genders. All these aspects need to be addressed and investigated to globally understand the role of neuroinflammation in AD and develop selective strategies.

## Hormones

Sex hormonal variations are a strongly debated risk factor for AD development and, as pointed out recently, they might interplay with other hormones in a refined modulation of neurocognitive functions. Different models have been proposed to study the peri-menopause influence on AD development. Marongiu suggested the accelerated ovarian failure as an optimal model to mimic the human process. This model could indeed be used to understand the molecular pathways implicated in sex hormone-related changes increasing AD risk. Rahman et al. underlined in their review the remarkable variety of pathophysiological conditions that are directly regulated by estrogen or that could be related to an alteration of its levels, like cardiovascular diseases (CVD). In this case, the increased risk of CVD is linked to an altered level of cholesterol, which is frequently observed in association with menopause. Indeed, estrogen seems to play a role in a variety of pathologies going from diabetes to depression, including infections and chronic inflammation. An aspect that should be particularly well-evaluated is the timing of a possible intervention in order to re-establish a hormonal balance. Evidences coming from recent studies are discussed and conclusions are pointing to the need of clinical trials and research investigations considering gender differences and leading to precision medicine approaches capable of analyzing the complexity of a patient clinical history. One of the gender differences that has been investigated concerns the consequences of chronic stress, which could be impacted by the activity of gonadal hormones (Rahman et al.). Indeed, AD has been proposed as a stress-related disorder by Canet et al.. In their up-to-date review, the authors evidenced how a dysregulation of the hypothalamic-pituitary-adrenal axis (HPA axis) has been observed in AD patients and could therefore represent a target for intervention. Molecules acting on the HPA axis hormones and/or receptors are already in clinical trials for other pathologies and the research for AD treatments could take advantage of such information.

## Neurovascular Unit

The neurovascular unit (NVU) is a functional brain unit composed my multiple cell types, including endothelial cells, smooth muscle cells, pericytes, and glial cells. The NVU is responsible for maintaining brain homeostasis by regulating permeability of the blood brain barrier (BBB), as well as clearance of unwanted products (such as Aβ) from the brain. Giannoni et al. emphasized in their review how the regulation of BBB permeability is of outmost importance for brain health, and its deregulation is associated with AD and related vascular dementias through multiple mechanisms, including auto-antibody responses and altered gut microbiome. The manuscript by Nizari et al. evaluated the effect of the loss of cholinergic innervation of components of the NVU in hippocampus and cortex, suggesting that cortical arteries are more affected by cholinergic denervation than hippocampal arteries, and pointing to a differential regulation of neurovascular responses in different brain areas. In regard to clearance mechanisms, Aldea et al. proposed through sophisticated mathematical modeling that localized contraction of smooth muscle cells generates the force that drives intramural periarterial drainage of Aβ and other soluble metabolites in the brain.

Circulating blood cells and vesicles may also contribute to BBB function and neurovascular regulation. Interestingly, Espinosa-Parrilla et al. reviewed the link between platelet aging and multiple neurodegenerative diseases including AD, discussing the role of platelets as drivers of protein dysfunctions, and the potential clinical significance of platelets and related miRNAs as peripheral biomarkers of neurodegenerative diseases.

## Multi-Interventions

Norwitz et al. highlight how AD might be the result of different dysregulations that interplay with each other in a complex and heterogenous matrix. In particular, they focus on the Wnt-signaling, α-synuclein and diabetes hypothesis, that might all contribute at different points and in different ways to the toxicity of the major AD hallmarks, Aβ and tau. In this context, AD therapies engaging simultaneously several molecular actors of the disease might achieve better efficacy in clinical trials. This multiplicity of action can be obtained by multi-target-directed ligands (MTDLs). In the present topic, Cuadrado-Tejedor et al. presented the compound CM-695, a dual inhibitor of histone deacetylase 6 (HDAC6) and phosphodiesterase 9 (PDE9). This MTDL demonstrated a therapeutic effect upon chronic administration to Tg2576 mice. In the same line, Hatat et al. developed an innovative molecule with triple activity of potential therapeutic interest against AD: –activation of serotonin type 4 (5-HT_4_) receptors, –inhibition of 5-HT_6_ receptors and –inhibition of acetylcholinesterase (AChE). Acute administration of this compound to mice prevented the memory deficits induced by scopolamine. Pleiotropic synergistic effects can also be demonstrated with molecules targeting α-secretase activity, such as acitretin. Indeed, dos Santos Guilherme et al. demonstrated that this molecule increased cerebrospinal fluid levels of interleukin 6 (IL6). Whether this effect is a direct effect of the molecule on an unknown target or is a consequence of retinoid control on inflammation has to be further investigated.

## Biomarkers

Facing the complexity and the heterogeneity of AD pathology, early, reliable, and easy to access biomarkers will be fundamental for effective therapies. Combining the current knowledge and routinely used biomarkers, such as Aβ and tau quantification and imaging, Younes et al. identified changepoints of these biomarkers far before the clinical symptoms. The future goal would be being able to point out individuals likely to progress to AD as they age. New biomarkers could help to develop an accurate prediction. One strategy could be to implement biomarkers tracking cellular metabolism such as nicotinamide adenine dinucleotide (NAD+). In this Research Topic, Grant et al. presented the fate of NAD+ and its metabolites following an intravenous infusion of NAD+ in a human cohort. As gut microbiota composition seems to be altered in AD patients and to have an impact on amyloid pathology (Cerovic et al.), another strategy to identify innovative biomarkers could be to follow GM composition. Would the huge amount of data collected by metagenomic analysis of bacterial taxa abundancy be informative to point out predictive, stratifying or prognostic biomarkers? This remains to be demonstrated. The success might come from the quantification of bacteria-derived metabolites such as bile acids or short chain fatty acids (SCFA).

## Concluding Remarks

This series of articles is representative of the complexity of AD pathology. It clearly underlines the importance of transdisciplinary studies that are linking multiple aspects of the pathology or, as pointed out in many reviews, of different subtypes of AD etiology and development. The sharing of pathological features with other diseases, although complex to analyze, may also open numerous treatment possibilities. For exemple, Deering Brose et al. suggest the use of hydroxyurea, an FDA-approved ribonucleotide reductase inhibitor currently prescribed to treat cancer, to ameliorate the cognitive deficits in AD. The use of repurposed drugs can notably accelerate clinical trials bringing to novel therapeutics through a relatively rapid protocol. The example of hydroxyurea, which is tested in a down syndrome mouse model but suggested for a broad range of neurodegenerative diseases (Deering Brose et al.), highlights how future drugs may be able to target pathways shared by multiple pathologies.

## Author Contributions

All authors listed have made a substantial, direct and intellectual contribution to the work, and approved it for publication.

## Conflict of Interest

The authors declare that the research was conducted in the absence of any commercial or financial relationships that could be construed as a potential conflict of interest.

